# Impact of Surgical Wait Time on Survival in Patients With Upper Urinary Tract Urothelial Carcinoma With Hydronephrosis

**DOI:** 10.3389/fonc.2021.698594

**Published:** 2021-07-05

**Authors:** Fangzheng Zhao, Nienie Qi, Chu Zhang, Ning Xue, Shuaishuai Li, Raorao Zhou, Zeyu Chen, Ruiqin Yao, Haitao Zhu

**Affiliations:** ^1^ Department of Urology, The Affiliated Hospital of Xuzhou Medical University, Xuzhou, China; ^2^ Department of Neurobiology, Xuzhou Key Laboratory of Neurobiology, Xuzhou Medical University, Xuzhou, China; ^3^ Department of Cell Biology and Neurobiology, Xuzhou Medical University, Xuzhou, China

**Keywords:** upper urinary tract urothelial carcinoma, hydronephrosis, surgical wait time, overall 5-year survival rate, cancer-specific survival (CSS)

## Abstract

**Background and Objectives:**

Due to the inevitability of waiting time for surgery, this problem seems to have become more pronounced since the outbreak of COVID-19, and due to the high incidence of preoperative hydronephrosis in upper urinary tract urothelial carcinoma (UTUC) patients, it is particularly important to explore the impact of preoperative waiting time and hydronephrosis on upper urinary urothelial carcinoma.

**Methods:**

316 patients with UTUC who underwent radical surgery at a high-volume center in China between January 2008 and December 2019 were included in this study. We retrospectively collected the clinicopathologic data from the medical records, including age, sex, smoking history, ECOG performance status (ECOG PS), body mass index (BMI), tumor location and size, number of lesions, T stage, N stage, surgical approach and occurrence of hydronephrosis, lymph node invasion, lymph node dissection, surgical margin, tumor necrosis, infiltrative tumor architecture, lymphovascular invasion and concomitant bladder cancer. Surgical wait time was defined as the interval between initial imaging diagnosis and radical surgery of UTUC. Hydronephrosis was defined as abnormal dilation of the renal pelvis and calyces due to obstruction of the urinary system. Firstly, all patients were divided into short-wait (<31 days), intermediate-wait (31-90 days) and long-wait (>90 days) groups according to the surgical wait time. The clinicopathological characteristics of each group were evaluated and the survival was compared. For patients with hydronephrosis, we subsequently divided them into two groups: short-wait (≤60 days) and long-wait (>60 days) groups according to the surgical wait time. Univariate and multivariate COX regression analysis were performed to evaluate the prognostic risk factor for patients with hydronephrosis.

**Results:**

A total of 316 patients with UTUC were included in this study with a median surgical wait time of 22 days (IQR 11-71 days). Of the 316 patients, 173 were classified into the short-wait group (54.7%), 69 into the intermediate-wait group (21.8%) and 74 into the long-wait group (23.5%). The median follow-up time for all patients was 43 months (IQR 28-67months). The median surgical wait times of the short-wait, intermediate-wait and long-wait group were12 days (IQR 8-17days), 42days (IQR 37-65days) and 191days (IQR 129-372days), respectively. The 5-year overall survival (OS) of all patients was 54.3%. The 5-year OS of short-wait, intermediate-wait and long-wait groups were 56.4%, 59.3% and 35.1%, respectively (P=0.045). The 5-year cancer-specific survival (CSS) of short-wait, intermediate-wait and long-wait groups were 65.8%, 70.9% and 39.6%, respectively (P=0.032). In the subgroup analysis, we divided 158 UTUC patients with hydronephrosis into short-wait group (≤60 days) and long-wait group (> 60 days), 120 patients were included in the short-wait group and 38 patients in the long-wait group. The median surgical wait times of the short-wait and long-wait group were 14days (IQR 8-28days) and 174days (IQR 100-369days), respectively. The 5-year OS of long-wait group was significantly lower than the OS of short-wait group (44.2% *vs.* 55.1%, P =0.023). The 5-year CSS of long-wait and short-wait group were 49.1% and 61.7%, respectively (P=0.041). In multivariate Cox regression analysis of UTUC patients with hydronephrosis, surgical wait time, tumor grade, pathological T stage, and tumor size were independent risk factors for OS and CSS. Lymph node involvement was also a prognostic factor for CSS.

**Conclusion:**

For patients with UTUC, the surgical wait time should be limited to less than 3 months. For UTUC patients with hydronephrosis, the OS and CSS of patients with surgical wait time of more than 60 days were relatively shorted than those of patients with surgical wait time of less than 60 days.

## Introduction

Upper urinary tract urothelial carcinoma (UTUC) is a relatively rare malignant tumor originating in the urethral epithelium. They can be located in the pyelocaliceal cavities and ureter. UTUC accounts for 5%-10% of all urothelial carcinomas in Western countries (1). A retrospective study concluded that the incidence of UTUC in the United States increased gradually from 1973 to 2005 (2). This situation is likely to continue as living conditions deteriorate and exposure to toxic factors increases. Although some studies have indicated the appropriate range of conservative or endoscopic treatment, radical nephroureterectomy (RNU) with bladder cuff resection remains the standard treatment for high-risk UTUC, regardless of tumor location (3).

Due to the aggressive nature of UTUC, urologists recommend that prompt surgical treatment is necessary for patients with a definite diagnosis (4). However, a certain preoperative waiting time is inevitable. The reasons for the delay of surgery include objective factors (including the capacity of the large-volume center, contraindications to surgery and patient tolerance) and subjective factors (patients’ attitudes towards the necessity of surgery). Since the outbreak of COVID-19, the situation of delayed surgery has become more obvious. In order to control the spread of the epidemic and treat infected patients to the greatest extent, foreign public health authorities and medical societies have suggested that all medical centers postpone or cancel non-emergency operations (5). Inevitably, patients may worry about the progression of the disease. The European Association of Urology (EAU) guidelines indicate that delayed surgery may increase the risk of progression of aggressive UTUC and recommend that patients should undergo radical surgery within 12 weeks of diagnosis (6).

Previous studies have suggested that preoperative hydronephrosis is an important prognostic factor in patients with UTUC, and hydronephrosis is often associated with higher pathological stage and poorer prognosis (7–9). In this study, we aimed to assess the appropriate surgical wait time for UTUC patients with hydronephrosis.

## Patients and Methods

316 patients with UTUC who underwent radical surgery at a high-volume center in China between January 2008 and December 2019 were included in this study. Patients with distant metastases and patients who received neoadjuvant chemotherapy or conservative treatment preoperatively were not included in this study. We retrospectively collected the clinicopathologic data from the medical records, including age, sex, smoking history, ECOG performance status (ECOG PS), body mass index (BMI), tumor location and size, number of lesions, T stage, N stage, surgical approach and occurrence of hydronephrosis, lymph node invasion, lymph node dissection, surgical margin, tumor necrosis, infiltrative tumor architecture, lymphovascular invasion and concomitant bladder cancer. Surgical wait time was defined as the interval between initial imaging diagnosis and radical surgery of UTUC. Hydronephrosis was defined as abnormal dilation of the renal pelvis and calyces due to obstruction of the urinary system. Hydronephrosis was confirmed by preoperative urological color Doppler ultrasonography, CT or MRI. The majority of UTUC patients were diagnosed accidentally by health checkup, and only a small number of patients admitted to hospital due to typical symptoms (such as gross hematuria and flank pain).

Firstly, all patients were divided into short-wait (<30 days), intermediate-wait (31-90 days) and long-wait (>90 days) groups according to the surgical wait time. The clinicopathological characteristics of each group were evaluated and the survival was compared. For patients with hydronephrosis, we subsequently divided them into two groups: short-wait (≤60 days) and long-wait (>60 days) groups according to the surgical wait time. Univariate and multivariate COX regression analysis were performed to evaluate the prognostic risk factor for patients with hydronephrosis. Surgical wait time (“ interval “) was included as a categorical variable in the COX regression analysis. The study was approved by the institutional review board from The Affiliated Hospital of Xuzhou Medical University. Written informed consent was obtained from the involving patient for the publication of this study. In order to confirm the original diagnosis, we invited an experienced urological pathologist to check all the pathological specimens again. The tumor stage was determined based on the American Joint Committee on Cancer (AJCC) staging system. The tumor grade was defined according to the 2004 World Health Organization (WHO) classification system. We invited an experienced urological radiologist and an experienced ultrasonologist to reexamine the preoperative imaging and ultrasound data of the patient to confirm the occurrence of hydronephrosis.

### Statistical Analysis

Continuous variables were compared by independent sample t-test and one-way analysis of variance test, and the χ2 test or Kruskal-Wallis test was used to evaluate categorical variables. Cumulative survival was estimated by the Kaplan-Meier curves. Independent prognostic factors were identified by univariate and multivariate analyses using the Cox proportional hazards model.A two-sided p-value of <0.05 was considered significant. All the statistical analyses were conducted using SPSS version 26.0 (IBM Corporation, Armonk, NY, USA).

## Results

A total of 316 patients with UTUC were included in this study, including 205 males (64.9%) and 111 females (35.1%). The median surgical wait time was 22days (IQR 11-71days). The median age of all patients was 69years (IQR 61-75years). There were 70 patients (22.2%) with a smoking history. The median body mass index is 22.6 (kg/m^2^) (IQR 20.1-25.2) (kg/m^2^). 223(70.6%) patients presented with hematuria and 158(50%) patients presented with hydronephrosis. In this study, 32 patients received adjuvant chemotherapy after surgery. The clinical characteristics, surgical data and pathological results of all patients were shown in **Table 1**. Of the 316 patients, 173 were classified into the short-wait group (54.7%), 69 into the intermediate-wait group (21.8%) and 74 into the long-wait group (23.5%). There were no significant differences among the three groups in age, sex, smoking history, ECOG performance status, median body mass index, hydronephrosis, tumor grade, tumor size, amount of lesions, pT stage, lymph node involvement, lymph node dissection, surgical approach, surgical margin, tumor necrosis, infiltrative tumor architecture, lymphovascular invasion and concomitant bladder cancer.

**Table 1:** Clinical characteristics of patients in the short-, intermediate-, and long-wait groups.

Varible	All patients (n = 316)	SWT (days) Short <31 (n = 173)	Intermidiate [31,90] (n = 69)	Long >90 (n = 74)	*P*
Age (yr)	69 (61-75)	69 (59-75)	69 (68-79)	68 (61-75)	0.376
Sex					0.352
Male	205	110 (34.8%)	42 (13.3%)	53 (16.8%)	
Female	111	63 (19.9%)	27 (8.5%)	21 (6.7%)	
Smoke					0.511
Yes	70	34 (10.8%)	17 (5.4%)	19 (6.1%)	
No	245	138 (43.7%)	52 (16.5%)	55 (17.5%)	
ECOG performance status					0.986
0	199	109 (34.6%)	43 (13.7%)	47 (14.9%)	
1	116	63 (19.9%)	26 (8.3%)	27 (8.6%)	
Median Body mass index (kg/m^2^)	22.6 (20.1-25.2)	22.5 (20.4-24.7)	22.4 (20.1-24.9)	22.8 (19.7-25.3)	0.120
Haematuria					0.000
(+)	223	95 (30.1%)	58 (18.4%)	70 (22.2%)	
(-)	91	77 (24.4%)	11 (3.5%)	3 (0.9%)	
Hydronephrosis					0.429
(+)	158	92 (29.1%)	33 (10.4%)	33 (10.4%)	
(-)	158	81 (25.6%)	36 (11.4%)	41 (13.1%)	
Tumor location					0.032
Renal pelvis	173	84 (26.6%)	40 (12.7%)	49 (15.5%)	
Ureter	143	89 (28.2%)	29 (9.2%)	25 (7.8%)	
Tumor grade					0.311
High	234	132 (41.8%)	52 (16.5%)	50 (15.8%)	
Low	81	40 (12.8%)	17 (5.4%)	24 (7.7%)	
Tumor size	3.5 (2.5-5)	3.5 (2.5-5.0)	3.5 (2.5-4.8)	3.0 (2.0-5.0)	0.949
Amount of lesions					0.137
Single	270	144 (45.6%)	64 (20.1%)	62 (19.6%)	
Mutiple	45	29 (9.2%)	5 (1.6%)	12 (3.9%)	
pT stage					0.310
≤pT1	87	51 (16.1%)	14 (4.4%)	22 (7.0%)	
pT2	111	54 (17.0%)	30 (9.5%)	27 (8.4%)	
pT3	97	57 (18.0%)	17 (5.3%)	23 (7.3%)	
pT4	21	11 (6.6%)	8 (3.4%)	2 (0.6%)	
Lymph node involvement					0.706
pN0	282	151 (47.8%)	64 (20.3%)	67 (21.1%)	
pN+	34	22 (7.0%)	5 (1.6%)	7 (2.2%)	
LND					0.861
Yes	81	46 (14.6%)	16 (5.1%)	19 (6.0%)	
No	235	127 (40.2%)	53 (16.8%)	55 (17.4%)	
Surgical approch					0.352
Open	67	37 (11.7%)	18 (5.7%)	12 (3.9%)	
Laparoscopy	249	136 (43.0%)	51 (16.1%)	62 (19.6)	
Surgical margin					1.000
Positive	0	0 ( 0.0%)	0 (0.0%)	0 (0.0%)	
Negative	316	173 (54.7%)	69 (21.8%)	74 (23.4%)	
Tumor necrosis					0.789
Yes	87	48 (15.2%)	17 (5.4%)	22 (7.0%)	
No	229	125 (39.6%)	52 (16.5%)	52 (16.5%)	
Infiltrative tumor					0.313
Architecture					
Yes	229	122 (38.6%)	55 (17.4%)	52 (16.5%)	
No	87	51 (16.1%)	14 (4.4%)	22 (7.0%)	
lymphovascular invasion					0.128
Yes	38	26 (8.2%0	4 (1.3%)	8 (2.5%)	
No	278	147 (46.5%)	65 (16.5%)	66 (20.9%)	
Complicated with bladder cancer					
Yes	20	10 (3.2%)	5 (1.6%)	5 (1.6%)	0.901
No	296	163 (51.6%)	64 (20.3%)	69 (21.8%)	

ECOG, Eastern Cooperative Oncology Group.

The median surgical wait time of the short-wait, intermediate-wait and long-wait groups were 12days (IQR 8-17days), 42days (IQR 37-65days), and 191days (IQR 129-372days), respectively. There was a significant difference in the incidence of hematuria between the intermediate- and long-term groups (P<0.001). The incidence of hydronephrosis was approximately 50% in all patients and there was no significant difference between the three groups (P=0.429).

**Figure 1:**
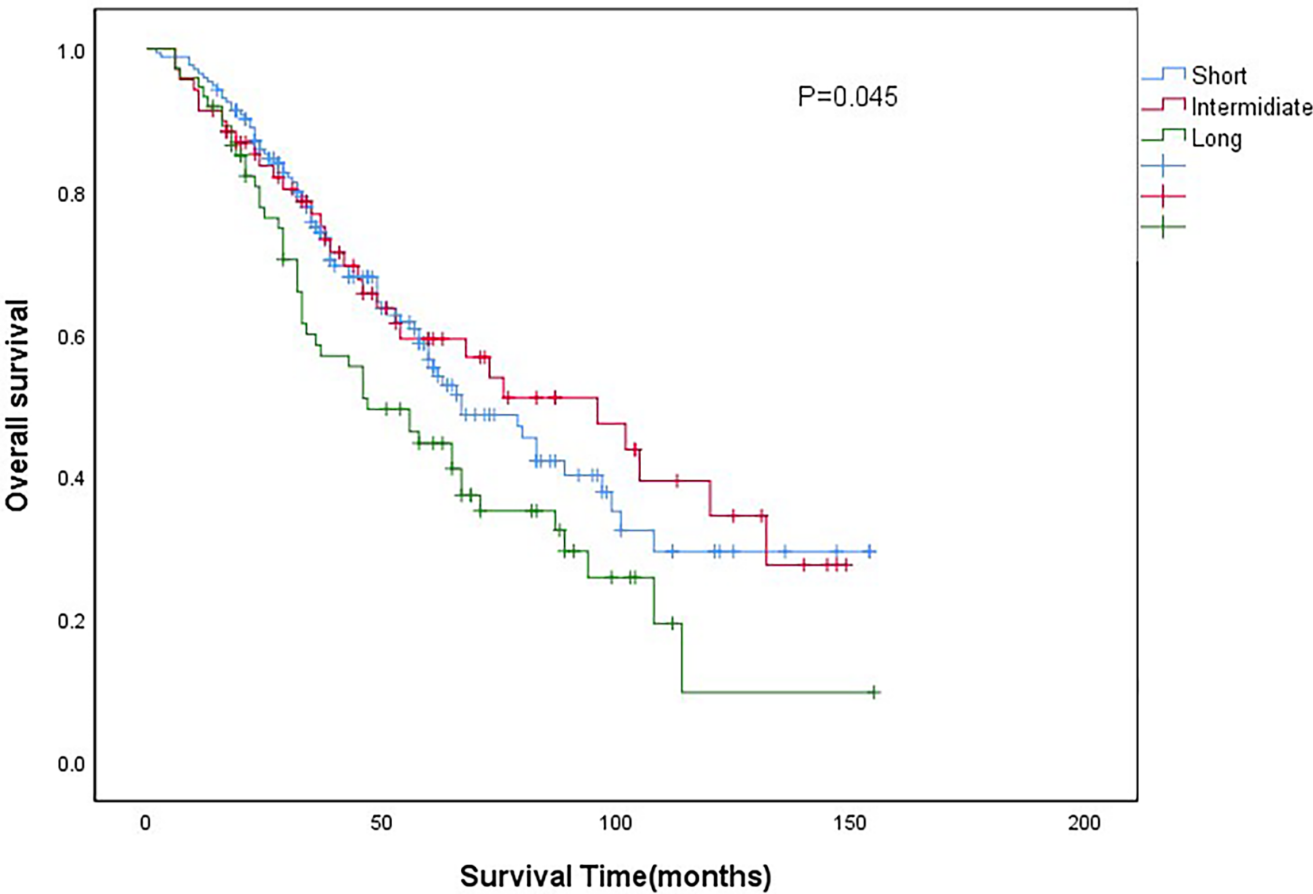
Comparison of overall survival curves between patients with different wait groups.

The median follow-up time for all patients was 43months (IQR 28-67months). The 5-year overall survival (OS) of all patients was 54.3%. The 5-year OS of short-wait, intermediate-wait and long-wait groups were 56.4%, 59.3% and 35.1%, respectively (P=0.045). The 5-year cancer-specific survival (CSS) of short-wait, intermediate-wait and long-wait groups were 65.8%, 70.9% and 39.6%, respectively (P=0.032). As shown in **Figure 1**, there was no significant difference in OS between the short-wait and intermediate-wait group. However, the OS of long-wait group was significantly shorted. After adjusting for gender, ECOG PS, histological type and pathological grade, we found that surgical wait time of more than 90 days was associated with a decrease in CSS or OS.

In the subgroup analysis, we divided 158 UTUC patients with hydronephrosis into short-wait group (≤60 days) and long-wait group (> 60 days), 120 patients were included in the short-wait group and 38 patients in the long-wait group. As shown in **Table 2**, the median surgical wait times of the short-wait and long-wait group were 14days (IQR 8-28days) and 174days (IQR 100-369days), respectively. There were no significant differences in age, sex, smoking history, ECOG performance status, median body mass index, tumor grade, tumor size, amount of lesions, pT stage, lymph node involvement, lymph node dissection and surgical approach between the two groups of UTUC patients with hydronephrosis. The incidence of hematuria in the long-wait group was significantly higher than that in the short-wait group (P<0.001).

**Table 2:** Clinical characteristics of patients in the short-, and long-wait groups.

Varible	All patients (n = 158)	SWT (days) Short ≤60 (n = 120)	Long >60 (n = 38)	*P*
Age (yr)	68 (61-74)	68 (61-74)	67 (60-73)	0.763
Sex				0.241
Male	91	66 (41.8%)	25 (15.8%)	
Female	67	54 (34.2%)	13 ( 8.2%)	
Smoke				0.269
Yes	28	19 (12.0%)	9 ( 5.7%)	
No	130	101 (63.9%)	29 (18.4%)	
ECOG performance status				0.755
0	99	76 (48.1%)	23 (14.6%)	
1	59	44 (27.8%)	15 ( 9.5%)	
Median Body mass index (kg/m^2^)	21.4 (19.3-23.5)	22.5 (20.4-24.7)	23.2 (19.5-25.2)	0.123
Haematuria				0.000
(+)	95	61 (38.6%)	34 (21.5%)	
(−)	63	59 (37.4%)	4 ( 2.5%)	
Tumor location				0.166
Renal pelvis	106	84 (53.2%)	22 (13.9%)	
Ureter	52	36 (22.8%)	16 (10.1%)	
Tumor grade				0.952
High	120	91 (57.5%)	29 (18.4%)	
Low	38	29 (18.4%)	9 ( 5.7%)	
Tumor size	3.5 (2.5-5.5)	3.5 (2.5-5.5)	3.5 (3.0-5.0)	0.886
Amount of lesions				0.199
Single	134	102 (64.6%)	32 (20.3%)	
Mutiple	24	18 (11.4%)	6 ( 3.7%)	
pT stage				0.866
≤pT1	36	28 (17.7%)	8 ( 5.1%)	
pT2	66	48 (30.4%)	18 (11.4%)	
pT3	45	35 (22.1%)	10 ( 6.3%)	
pT4	11	9 ( 5.7%)	2 ( 1.3%)	
Lymph node involvement				0.867
pN0	138	105 (66.4%)	33 (20.9%)	
pN+	20	15 ( 9.5%)	5 ( 3.2%)	
LND				0.490
Yes	51	37 (23.4%)	14 ( 8.9%)	
No	107	83 (52.5%)	24 (15.2%)	
Surgical approach				0.488
Open	43	31 (19.6%)	12 ( 7.6%)	
Laparoscopy	115	89 (56.3%)	26 (16.5%)	

ECOG, Eastern Cooperative Oncology Group.

**Figure 2:**
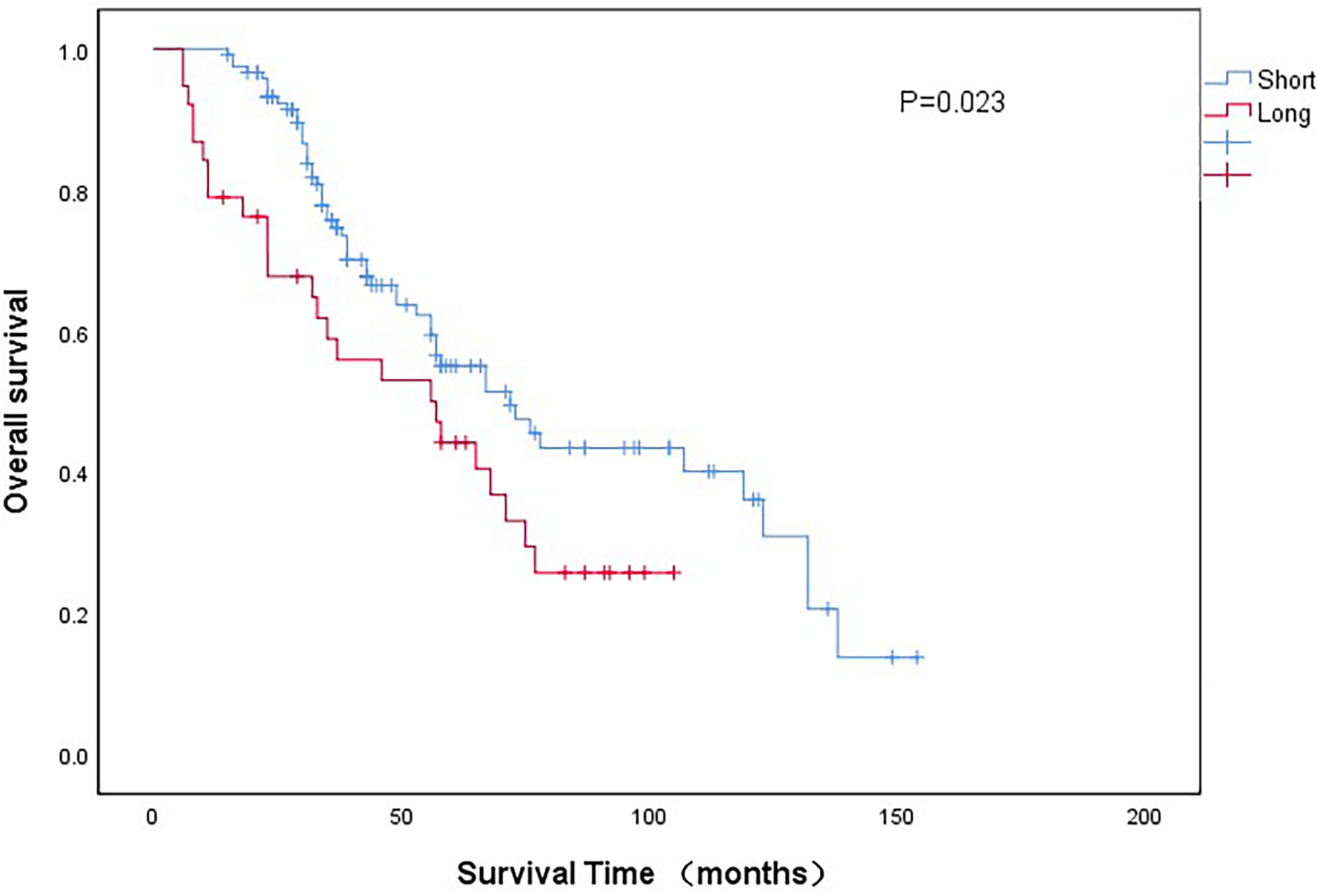
Comparison of overall survival curves between patients with different wait groups.

As shown in **Figure 2**, the 5-year OS of long-wait group was significantly lower than the OS of short-wait group (44.2% *vs* 55.1%, P =0.023). The 5-year CSS of long-wait and short-wait group were 49.1% and 61.7%, respectively (P=0.041). In multivariate Cox regression analysis of UTUC patients with hydronephrosis, surgical wait time, tumor grade, pathological T stage, and tumor size were independent risk factors for OS and CSS. Lymph node involvement was also a prognostic factor for CSS (**Table 3**).

**Table 3:** Multivariable Cox model for cancer-specific survival and overall survival.

Variables	Cancer-specific survival		Overall survival	
	HR (95% CI)	*P* value	HR (95% CI)	*P* value
**Age**	1.02 (0.99-1.04)	0.160	1.01 (0.98-1.04)	0.503
**Sex**	1.06 (0.67-1.66)	0.818	0.82 (0.50-1.36)	0.450
**Smoke**	0.93 (0.53-1.64)	0.804	1.15 (0.63-2.12)	0.650
**Interval**	1.74 (1.07-2.82)	0.026	2.05 (1.20-3.50)	0.009
**ECOG PS**	1.22 (0.77-1.94)	0.388	1.02 (0.61-1.71)	0.952
**Haematuria**	1.06 (0.67-1.67)	0.802	1.02 (0.58-1.79)	0.960
**Surgical approach**	0.94 (0.59-1.50)	0.782	1.07 (0.65-1.79)	0.784
**LND**	1.40 (0.88-2.23)	0.153	1.58 (0.94-2.67)	0.085
**Tumor Grade**	3.30 (1.69-6.43)	0.000	2.72 (1.30-5.69)	0.008
**Tumor Location**	0.93 (0.58-1.48)	0.747	0.85 (0.50-1.45)	0.559
**Amount of lesions**	0.91 (0.49-1.69)	0.776	1.29 (0.65-2.57)	0.463
**pT Stage**	1.62 (1.24-2.11)	0.000	1.38 (1.02-1.87)	0.037
**Lymph node involvement**	1.71 (1.20-2.42)	0.003	1.25 (0.83-1.89)	0.281
**Tumor Size**	1.13 (1.05-1.22)	0.001	1.10 (1.01-1.19)	0.032

ECOG, Eastern Cooperative Oncology Group.

## Discussion

Urologists generally believe that surgery should be performed as soon as possible after diagnosis for patients with UTUC (4). However, objective factors such as preoperative medical evaluation, limitations in the health care system and the capacity of large-volume centers inevitably lead to the delay of surgery. In addition, some medical factors may also postpone the timing of radical surgery, including the introduction of neoadjuvant chemotherapy and ureteroscopy biopsy. In China, the main factors that lead to surgical delay include patients’ resistance to radical surgery, patients’ ability to pay and short-term intolerance to surgery due to underlying diseases. For the vast majority of patients in the study, radical surgery is performed as soon as possible once the diagnosis is confirmed. This is also the reason for the large proportion of patients in the short waiting group. However, the diagnosis may not be clear through imaging examination for some patients. They often require ureteroscopic biopsy to confirm the diagnosis prior to radical surgery. Although some patients hope to receive radical surgery as soon as possible, they need to be transferred to internal medicine for treatment due to surgical contraindications (such as cardio-cerebrovascular accidents and poor respiratory function). These are also the main reasons for delayed surgery. Waiting for surgery can be a great anxiety for patients with little medical knowledge (10). They may worry that the disease may progress while waiting for surgery and the prognosis may be affected (11). This problem seems to be more pronounced during COVID-19, so it is particularly important to study the effect of surgical wait time on cancer prognosis (12).

Although many scholars have evaluated the relationship between surgical wait time and the prognosis of UTUC (13–15), there are different results due to different inclusion criteria and study methods, and no consensus has been reached yet. The EAU guideline on UTUC showed that for patients with invasive UTUC, surgical delays increase the risk of disease progression. The guideline recommended that the time between diagnosis and surgery should be limited to less than 12 weeks (6). Consistent with the result of EAU guideline, we found that the OS and CSS of patients with UTUC who waited more than 3 months before surgery were significantly lower than those of patients who waited less than 3 months before surgery.

Prolonged surgical wait time may increase the rate of distant micrometastases. Waldert et al. retrospectively analyzed 187 UTUC patients who underwent radical surgery and concluded that surgical wait time of more than 3 months was often related to more advanced tumor stages and higher pathological grades (16). Similar results were seen in bladder cancer. In a grouping analysis of 441 patients who underwent radical cystectomy for muscle-invasive bladder carcinoma, a delay of more than 12 weeks in surgical treatment was associated with an increased risk of disease-specific and all-cause mortality (17). However, some scholars have expressed different opinions on this issue. In another study, cancer-specific and relapse-free survival (RFS) in 138 UTUC patients did not differ significantly between the early and delayed groups. However, in a subgroup analysis of ureteral urothelial carcinoma, the author identified a one-month delay in surgery as an independent prognostic factor for CSS and RFS. This result may be related to the anatomical differences between the ureter and the renal pelvis (18–20). Compared to renal parenchyma and perirenal fat, the ureteral wall is relatively thin and cannot act as a protective barrier for tumor. There are abundant blood vessels and lymphatics in the outer layer of the ureter, which is prone to distant metastasis. Nison L et al. evaluated the effect of delayed surgery on tumor prognosis in UTUC patients due to preoperative ureteroscopy (21). They found that although the implementation of preoperative diagnostic ureteroscopy delayed the time of RNU, there were no significant differences in CSS, RFS and metastasis-free survival. In another study, Haddad M. et al. divided 51 UTUC patients into immediate RNU group and delayed RNU group (after conservative treatment), and compared the pathological results of the two groups (22). They found that there was no significant difference in final pathological stage and grade between the two groups.

As UTUC is a rare and highly aggressive malignancy, it is helpful for clinicians to identify the associated risk factors. A number of factors have been generally recognized as prognostic factors, including preoperative risk factors (such as tobacco exposure, tumor location and multi-focality, American Society of Anesthesiology score) and postoperative risk factors (such as tumor stage and grade, lymph node involvement and lymphovascular invasion) (23–25). Previous studies have indicated that hydronephrosis is a risk factor for UTUC (7, 9, 26). In our subgroup analysis of patients with hydronephrosis, the OS and CSS of patients with surgical wait time of more than 60 days were relatively shorted than those of patients with surgical wait time of less than 60 days. We believe that the results may be mainly related to the following factors. Firstly, hydronephrosis may increase the pressure of the renal pelvis and ureter, leading to dilatation and thinning of the canal wall, thus making it easier for tumor cells to invade peripherally (27). Secondly, the increased pressure in the renal pelvis caused by hydronephrosis leads to ipsilateral renal function impairment and further aggravates the burden on the contralateral kidney (28). Finally, elevated pressure in the renal pelvis and ureteral wall may lead to ischemic changes in surrounding tissues thus inducing the expression of hypoxia-inducible factor-1a (HIF-1a), which may be involved in tumor growth and new blood vessels (29). Our analysis also showed that surgical wait time, tumor grade, pathological T stage, and tumor size are independent prognostic factors for UTUC patients with hydronephrosis.

In this study, a total of 23 cases of urothelial carcinoma with histological variants were identified and micropapillary carcinoma and squamous cell carcinoma were the predominant subtypes. Due to the limited data, they were not included in COX regression analysis. A previous study found that the incidence of histologic variation in UTUC was approximately 10%. Micropapillary and sarcomatoid variations may lead to poor oncology outcomes (30). Another study also suggested that the micropapillary variation often predicts a poor biological behavior in invasive urothelial carcinoma of the bladder (31). This phenomenon may be related to the overexpression of HER2 protein, but this result needs to be confirmed in a larger, multi-institutional study.

We chose the waiting time “cut-offs” in the overall population and in patients with hydronephrosis based on the following reasons. Although the majority of patients in our study waited less than 30 days for surgery, many patients waited longer than 90 days before surgery. A 3-month delay in radical cystectomy for muscle-infiltrating bladder cancer increases the risk of progression and cancer-specific mortality (17). We wanted to know if this applies to UTUC. Therefore, we divided all patients into three groups: short wait group (<31 days), intermediate wait group (31-90 days) and long wait group (> 90 days). Previous studies have suggested that hydronephrosis is a prognostic factor for UTUC (7, 9, 26). Therefore, we divided patients with hydronephrosis into a short waiting group (≤60 days) and a long waiting group (> 60 days) to investigate whether hydronephrosis would further shorten the “90-day” surgical safety window. Based on our research, we think that surgery delays > 60 days for UTUC patients with hydronephrosis may adversely affect the prognosis. Therefore, it is recommended to arrange radical surgery as soon as possible for these patients.

The present study has some limitations. In the first place, this study is a single-center, retrospective study, selection bias is unavoidable. In order to reduce the selection bias, once the preoperative examination is completed and the surgical contraindications are eliminated, we will arrange the surgery soon. Our hospital is a tertiary hospital with more than 4000 beds. All of the UTUC patients are treated equally. The patient’s surgical schedule will not be subject to the conflict of the operation day. In the second place, the follow-up time of this study was long (up to 10 years), and there were certain losses in the process of data collection. In addition, in order to avoid the interference of subjective factors on surgical wait time, radical surgery will be performed once UTUC is diagnosed in our hospital. Patients with distant metastases and patients who received neoadjuvant chemotherapy or conservative treatment preoperatively in other hospital were not included in this study. Therefore, we could not make reasonable recommendations for these patients. Finally, since there is no standard for the scope of lymph node dissection, it is often decided by surgeons according to their surgical experience and no effective data statistics can be formed. The relationship between prolonged surgical wait time and UTUC patients with hydronephrosis needs to be further studied.

## Conclusion

For patients with UTUC, the surgical wait time should be limited to less than 3 months. For UTUC patients with hydronephrosis, the OS and CSS of patients with surgical wait time of more than 60 days were relatively shorted than those of patients with surgical wait time of less than 60 days.

## Data Availability Statement

The raw data supporting the conclusions of this article will be made available by the authors, without undue reservation.

## Ethics Statement

The studies involving human participants were reviewed and approved by The Affiliated Hospital of Xuzhou Medical University. Written informed consent for participation was not required for this study in accordance with the national legislation and the institutional requirements.

## Author Contributions

FZ, NQ, and CZ conceived the study, participated in its design, collected the data, performed the statistical analysis, and drafted the manuscript. NX, SL, RZ and ZC participated in its data and manuscript proofreading. RY and HZ participated in its design, and helped to draft the manuscript. All authors contributed to the article and approved the submitted version.

## Funding

This work was supported by the 2018 Doctoral Project for Innovation and Entrepreneurship of Jiangsu Province and the Natural Science Foundation Youth Project of Jiangsu Province (BK20190989).

## Conflict of Interest

The authors declare that the research was conducted in the absence of any commercial or financial relationships that could be construed as a potential conflict of interest.
